# Metformin Induces Apoptosis and Alters Cellular Responses to Oxidative Stress in Ht29 Colon Cancer Cells: Preliminary Findings

**DOI:** 10.3390/ijms19051478

**Published:** 2018-05-16

**Authors:** Paola Sena, Stefano Mancini, Marta Benincasa, Francesco Mariani, Carla Palumbo, Luca Roncucci

**Affiliations:** 1 Department of Biomedical, Metabolic and Neurosciences, Section of Human Morphology, University of Modena and Reggio Emilia, Policlinico, Via Del Pozzo 71, I-41125 Modena, Italy; paola.sena@unimore.it (P.S.); marta.benincasa@unimore.it (M.B.); carla.palumbo@unimore.it (C.P.); 2 Department of Diagnostic and Clinical Medicine, and Public Health, University of Modena and Reggio Emilia, Policlinico, Via Del Pozzo 71, I-41125 Modena, Italy; mancini77@cloud.com (S.M.); francesco.mariani@unimore.it (F.M.)

**Keywords:** colorectal cancer cells, metformin, apoptosis, oxidative stress

## Abstract

Accumulating evidence suggests that metformin, used as an antidiabetic drug, possesses anti-cancer properties. Metformin reduced the incidence and growth of experimental tumors in vivo. In a randomized clinical trial among nondiabetic patients, metformin treatment significantly decreased the number of aberrant crypt foci compared to the untreated group with a follow-up of 1 month. In our study, HT29 cells were treated with graded concentrations of metformin, 10 mM/25 mM/50 mM for 24/48 h. We performed immunofluorescence experiments by means of confocal microscopy and western blot analysis to evaluate a panel of factors involved in apoptotic/autophagic processes and oxidative stress response. Moreover, HT29 cells treated with metformin were analyzed by a flow cytometry assay to detect the cell apoptotic rate. The results demonstrate that metformin exerts growth inhibitory effects on cultured HT29 cells by increasing both apoptosis and autophagy; moreover, it affects the survival of cultured cells inhibiting the transcriptional activation of Nuclear factor E2-related factor 2 (NRF-2) and nuclear factor-kappa B (NF-κB). The effects of metformin on HT29 cells were dose- and time-dependent. These results are very intriguing since metformin is emerging as a multi-faceted drug: It has a good safety profile and is associated with low cost and might be a promising candidate for the prevention or the treatment of colorectal cancer.

## 1. Introduction

Colorectal cancer (CRC) is still a major neoplasm [1], and its prevalence and mortality have a high impact on human health [2]. A paradigm shift from surveillance and early detection of cancer or adenomas to new preventive strategies, including chemoprevention, is necessary to lower the burden of this disease. Several large epidemiologic and clinical studies have evaluated the possible effects of more than 200 agents, including fibers, calcium, and nonsteroidal anti-inflammatory drugs such as 5-aminosalicylic acid and selective cyclooxygenase-2 (COX-2) inhibitors, in preventing CRC development [3]. Nonsteroidal anti-inflammatory drugs, especially COX-2 inhibitors alone or in combination, have shown the most promise for CRC risk reduction [4], but an increased risk of serious cardiovascular events associated with COX-2 inhibitor use has been reported [5,6]. Thus, novel drugs that would be both safe and effective are needed for CRC prevention. CRC is associated with lifestyle-related diseases such as diabetes and obesity [7,8,9,10]; these conditions might represent new targets for CRC chemoprevention. Metformin (1, 1-dimethylbiguanide hydrochloride) is a biguanide derivative that has been extensively used for treating diabetes mellitus [11]. It decreases basal glucose output by suppressing gluconeogenesis and glycogenolysis in the liver, and by increasing glucose uptake in muscle tissue. Because metformin does not directly stimulate insulin secretion, the risk of hypoglycemia associated with its use is lower than that associated with the use of other oral antidiabetic drugs [12]. The mechanism of action of metformin involves liver kinase B1–dependent activation of AMP-activated protein kinase [13]. Patients with type 2 diabetes taking metformin might be at a lower risk of cancer (including CRC), compared with those who do not take metformin [14,15]. This evidence suggests that metformin might be a candidate agent for the chemoprevention of CRC in diabetic patients. In a number of preclinical studies, metformin reduced cell proliferation, induced apoptosis, caused cell cycle arrest, and reduced incidence and growth of experimental tumors in vitro and in vivo [16,17]. Some reports also indicate that metformin improved the response of human breast tumor xenografts to conventional chemotherapy by eradicating cancer stem cells in the tumor [18,19]. HT-29 is a human colorectal adenocarcinoma cell line with epithelial morphology and represents a xenograft tumor model for colorectal cancer. Nuclear factor E2-related factor 2 (NRF-2) is a transcription factor that controls the expression of a large pool of antioxidant and cytoprotective genes regulating the cellular response to oxidative and electrophilic stress. Mutations in the *NRF-2* gene, common in cancer cells, could help tumor cells to survive, and might be associated with poor survival of cancer patients. Previous studies have shown that the NRF-2 signaling pathway is abnormally activated in CRC. NF-κB plays a major role in linking inflammation to cancer development through its ability to upregulate several inflammatory and tumor promoting cytokines, such as IL-6, IL-1α, and Tumor Necrosis Factor α (TNFα), as well as genes like *BCL2* and *BCLXL*. Furthermore, NF-κB plays an important role in type 2 diabetes mellitus (T2DM), as obesity activates the transcription factor NF-κB, which increases the risk for T2DM. Collectively, NF-κB could be considered as the matchmaker between inflammation, inflammatory bowel diseases (IBD), cancer, and diabetes [20]. The present study aims to assess whether metformin has the potential to suppress the growth of colorectal cancer cells and to evaluate its role in affecting the NRF-2/NF-κB pathways.

## 2. Results

### 2.1. Metformin Suppresses the Proliferation of HT29 Cells in a Dose- and Time-Dependent Manner

In order to examine whether metformin affects human colorectal cancer cell proliferation, we investigated the effect of the drug on the cancer cell line HT29 cells. Cells were grown in 10% fetal bovine serum (FBS) and treated with increasing amount of metformin (0, 10, 25, and 50 mmol/L). The immunofluorescence analysis was performed after the addition of the agents for 24 and 48 h, to characterize the time-dependent impacts of metformin.

**Figure 1:**
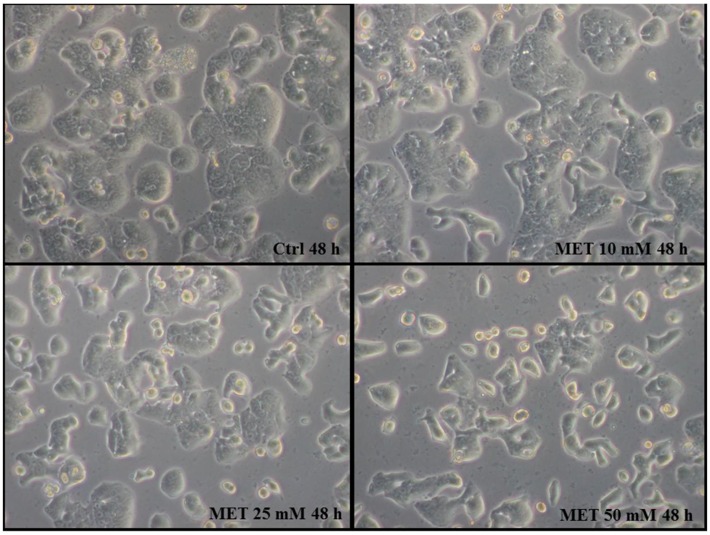
Inverted microscope image of HT29 cells treated with graded concentrations of metformin, 10 mM/25 mM/50 mM for 24 h and 48 h; and of untreated cells (CTRL). The phenotype of HT29 cells treated with metformin was preserved, although cell adhesion to the culture plates caused some changes in the characteristic rounded shape of untreated HT29 cells. Magnification 40×.

The first observation was made using an inverted microscope and raised concerns about the eventual changes of some features (viability, adhesion, and morphology) of the treated cells compared to controls; as shown in Figure 1 the phenotype of HT29 cells treated with metformin was preserved, although the cell adhesion to the culture plates implied some changes in the characteristic rounded shape of untreated HT29 cells Figure 1.

This modification of cell morphology is probably due to the partial loss of plasma membrane attachment, these features are characteristic of the earliest phases of the apoptotic process. Cell counting showed that metformin caused a strong anti-proliferative effect at a dose of 10 mM for 24 h; the effect was slight, but significant anti-proliferative action was observed with higher doses for 24 h or 48 h. The counting obtained was the following, 43%, 52%, and 65% inhibition for 10 mM, 25 mM, and 50 mM, respectively, compared with controls after 24 h treatment; 45%, 61%, and 68% inhibition for 10 mM, 25 mM, and 50 mM, respectively, compared with controls after 48 h treatment.

To confirm these preliminary data, immunofluorescence experiments, coupled with confocal analysis, were performed using a panel of antibodies to determine the effect of metformin treatment on the expression profile of autophagy/apoptosis or proliferation markers.

**Figure 2:**
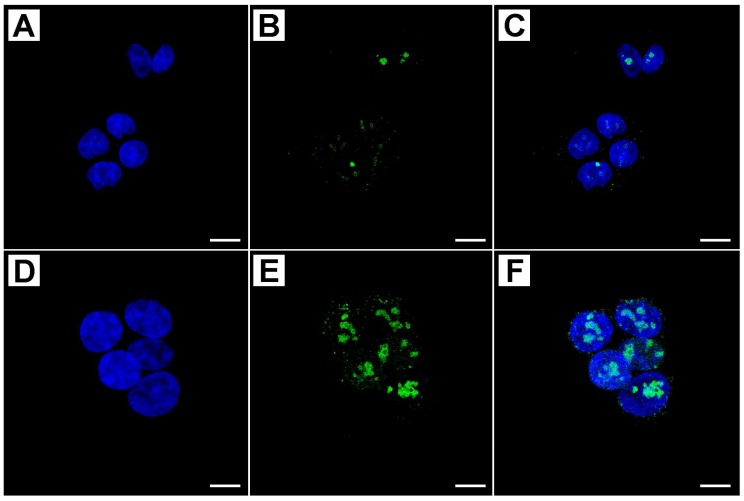
Confocal analysis of Ki-67 protein in treated and untreated cells with graded concentrations of metformin (blue: DAPI; Green: Ki-67; (**C**,**F**): merge). Cells that were treated with 10 mM MET for 48 h showed a weak immunostaining at the nuclear level (**A**–**C**), whereas untreated cells resulted very marked (**D**–**F**). Scale bar = 8 µm.

The Ki-67 protein was used as a marker for cell proliferation and the results from the immunofluorescence analysis Figure 2, and showed that metformin significantly downregulates the expression of Ki-67 and nuclear localization in a time- and dose-dependent manner.

The treatment at 48 h with MET 50 mM caused a maximum decrease of 45% in the proliferation index, as reported in Table 1, which collects data of semi-quantitative evaluation of immunostaining intensity (Immunofluorescence Intensity Score: IFIS).

Furthermore, immunofluorescence analysis was conducted using apoptotic and autophagic specific markers in order to determine whether the inhibitory effect of metformin on colorectal cancer cells was associated with triggering programmed cell death or autophagy.

**Figure 3:**
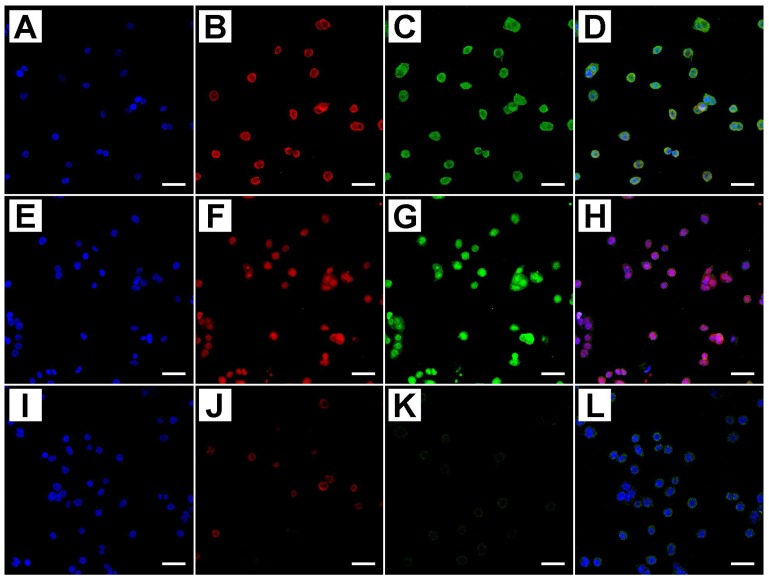
Confocal analysis of PARP-1 and Caspase-3 active proteins in treated and untreated cells with different concentrations of metformin (blue: DAPI; Red: PARP-1 Green: Caspase-3 active; (**D**,**H**,**L)**: merge). Cells that were treated with 10 mM MET for 24 h showed a strong immunostaining for both proteins (**A**–**D**), as well as cells treated with 25 mM MET for 24 h (**E**–**H**). Untreated cells showed a significant decrease in PARP-1 and Caspase-3 active protein expression (**I**–**L**). Scale bar = 15 µm.

Using these techniques, we evaluated both qualitatively and quantitatively Cleaved PARP-1, APAF-1, Caspase-3, and MAPLC3 protein expression. Figure 3 shows the co-immunostaining of Cleaved PARP-1 and Caspase-3.

Cleaved PARP-1 antibody detects endogenous levels of the large fragment (89 KDa) of the human protein resulting from cleavage of the native protein and does not recognize the full length PARP-1 or other isoforms. Cleaved PARP-1 was detectable in the nucleus of treated HT-29 cells; however, it is not appreciable in untreated cells Figure 3K. Some representative staining patterns are shown in Figure 3A–D where nuclear labeling of apoptotic cells is evident, as revealed by DAPI staining. Caspase-3 was aggregated in small clumps distributed in the cytoplasm of cultured treated cells, both proteins showed an increased expression pattern related to the dose and time of metformin treatment, as shown in Figure 3A–H. Untreated cells were negative for immunostaining Figure 3I–L.

**Figure 4:**
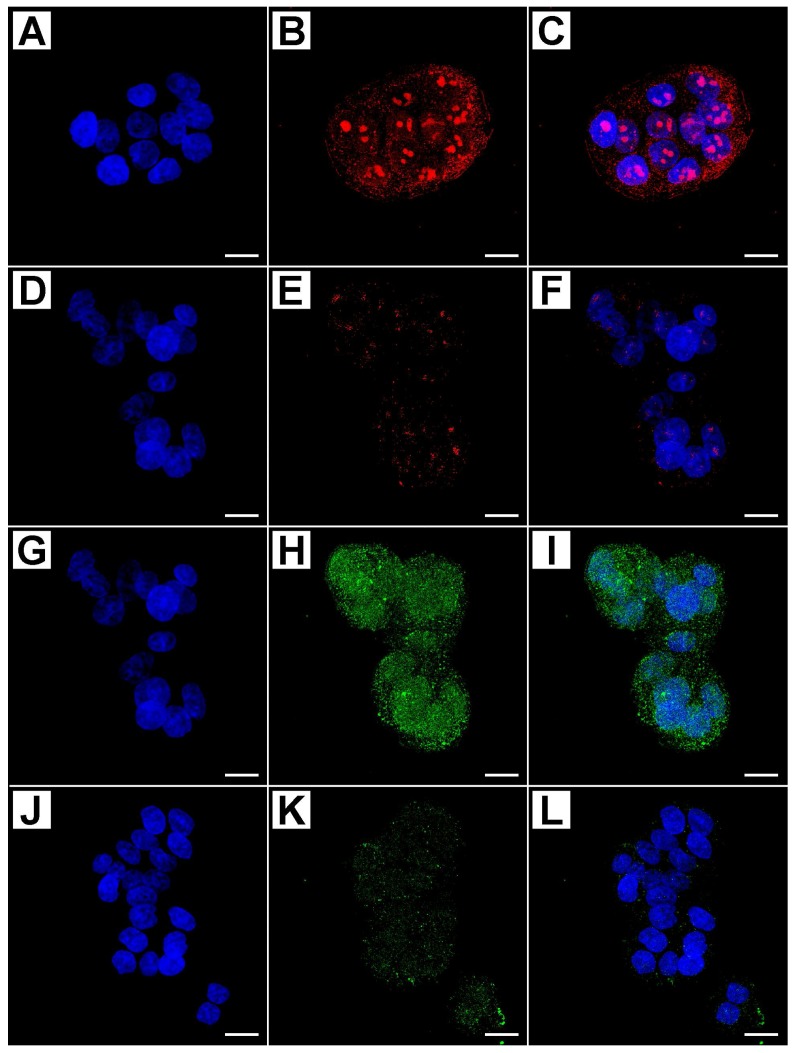
Confocal analysis of APAF-1 and MAPLC3 proteins in treated and untreated cells with different concentrations of metformin (Blue: DAPI; Green: MAPLC3; Red: APAF-1; (**C**,**F**,**I**,**L**): merge). In treated cells with 50 mM MET for 48 h, APAF-1 showed a diffuse or granular staining pattern at the nuclear level (**A**–**C**), while in untreated cells nuclear expression was barely detectable (**D**–**F**). In treated cells with 50 mM MET for 48 h MAPLC3 protein there were two distinct autophagic patterns: A diffuse finely and granular reactivity dispersed in the cytoplasm, or a rounded densely stained material, probably enclosed within a cytoplasmic vacuole that accumulates prevalently around the nucleus (**G**–**I**); untreated cells were very weakly marked (**J**–**L**). Scale bar = 10 µm.

Figure 4 shows the immunostaining of APAF-1 and MAPLC3.

The staining patterns of the first protein varied from diffuse to granular in the nucleus of treated cells; on the other hand, cells expressing MAPLC3 protein showed two distinct autophagic patterns: diffuse fine and granular reactivity was dispersed in the cytoplasm, or a rounded densely stained material, which was probably enclosed within a cytoplasmic vacuole that accumulates prevalently around the nucleus (Figure 4G–I).

The dense rounded autophagic vacuoles were well recognizable in cells treated with higher doses and for longer time; such structures varied in size and density, but usually formed coarse, rather than fine, granules. Untreated cells showed a weak marking for both proteins Figure 4D–F,J–L.

The semiquantitative evaluation of immunostaining intensity, reported as the Immunofluorescence Intensity Score (IFIS) in Table 1, showed that the level of cleaved PARP-1, Caspase-3, APAF-1, and MAPLC3 proteins had an increasing trend in a dose- and time-dependent manner, with statistical significance of the different expression among the groups of treated cells, and with respect to untreated cells.

**Figure 5:**
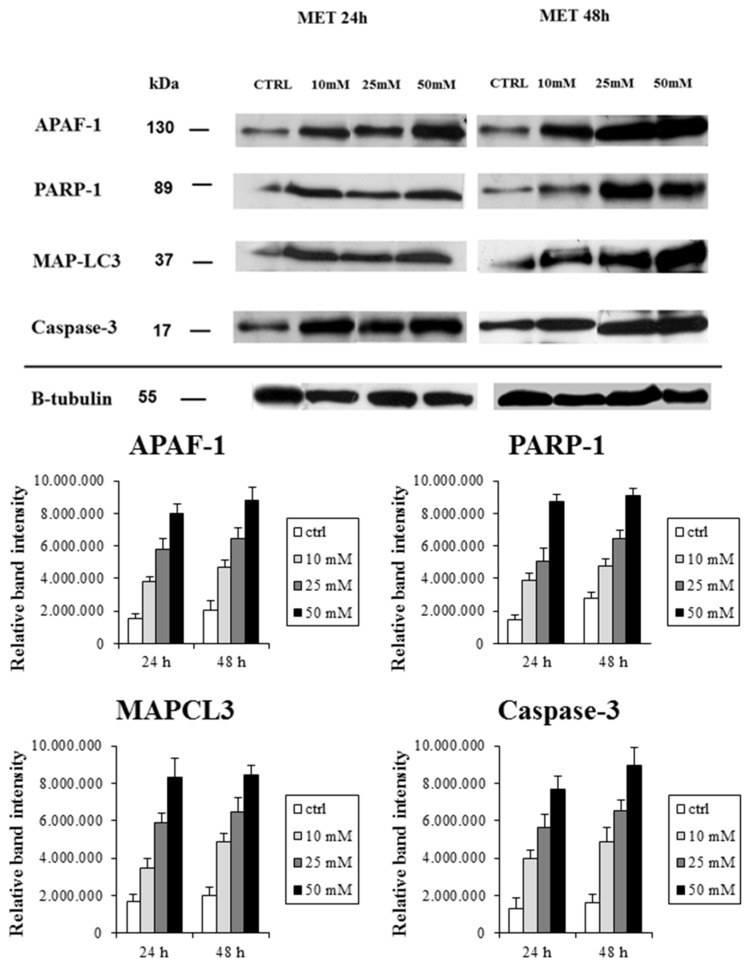
Western blot analysis of HT29 cells treated with graded concentrations of metformin, 10 mM/25 mM/50 mM for 24 h and 48 h; and of untreated cells (CTRL), using anti-APF-1, PARP-1, MAPLC3, and Caspase-3 antibodies. Mean densitometric data of APAF-1, PARP-1, MAPLC3, and Caspase-3 expressions were analyzed using NIH Image J software. *p* < 0.05 between all group pairs.

Moreover, to corroborate the findings of the morphological evaluation previously described, cell lysates of cultured treated and untreated cells were subjected to western blot analysis. As shown in Figure 5, protein bands immunopositive for APAF-1, Cleaved PARP-1, MAPLC3, and caspase-3 were clearly evident in treated cells.

Lysates of untreated cells generally yielded only a faint band for all these markers. Densitometric analysis and normalization (with equal amounts of protein loading) of the immunoreactivity signals from protein extracts of treated and untreated cells showed that the expression pattern had an increasing trend related to the dose- and time- of treatment. Western blotting and densitometric analysis were performed in triplicate; the quantification of protein expressions between different groups of treatment and respect to the untreated group achieved statistical significance.

### 2.2. Metformin Alters NRF-2 and NF-κB Expression in HT29 Cells in a Dose- and Time-Dependent Manner

**Figure 6:**
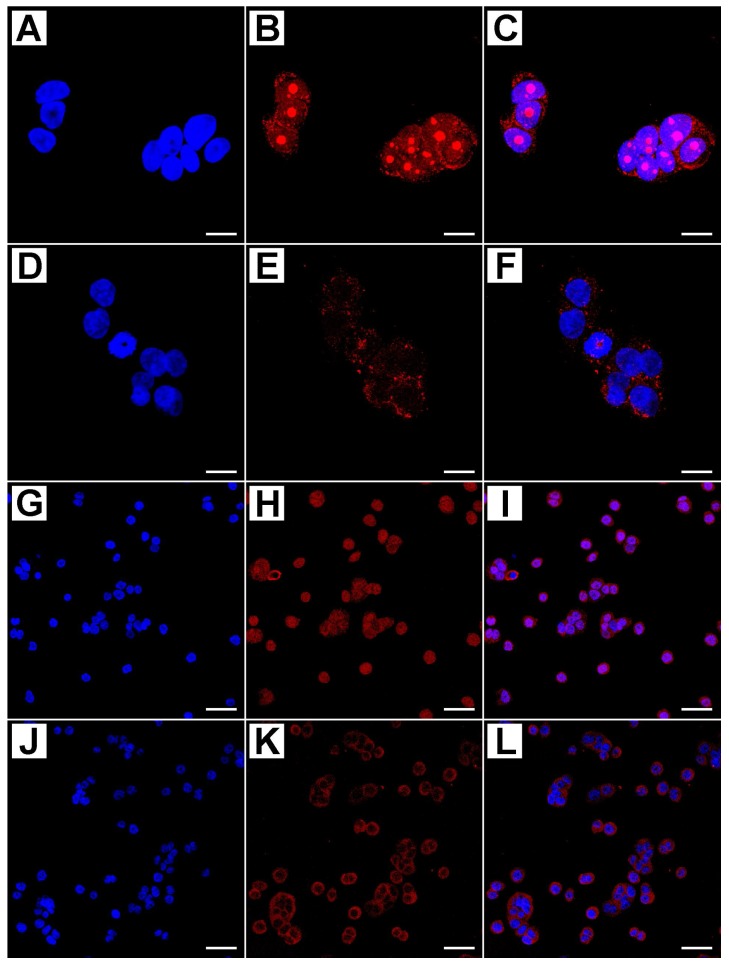
Confocal analysis of NRF-2 and NF-κB proteins in treated and untreated cells with graded concentrations of metformin (Blue: DAPI; Red: NRF-2, and NF-κB; (**C**,**F**,**I**,**L**): merge). NRF-2 clearly showed a strong nuclear reactivity in untreated cells (**A**–**C**), whereas in cells treated with 25 mM MET for 24 h the reactivity was finely dispersed only in the cytoplasmic compartment (**D**–**F**). NF-κB showed a significant translocation into the nucleus in untreated cells with 25 mM MET for 24 h (**G**–**I**), while in treated cells the protein was scattered in the cytoplasm (**J**–**L**). Scale bar = 10 µm.

Confocal analysis was conducted on HT29 cells treated with increasing amount of metformin (0, 10, 25, and 50 mmol/L), as described above, to analyze the expression pattern of NRF-2 and NF-κB proteins. The results demonstrated that metformin affects survival of cultured cells inhibiting the transcriptional activation of NRF-2 and NF-κB. In Figure 6A–C the presence of NRF-2 at the nuclear level in untreated cells is clearly evident, as a consequence of the translocation of the transcriptional factor into the nucleus; in particular, nucleolar domains appeared strongly marked Figure 6B.

Treated cells showed a well detectable presence of this protein in the cytoplasm, while the nuclei were slightly marked Figure 6D–F. The effects of metformin on HT29 cell were dose- and time-dependent, because there was a significant change in the parameters analyzed after 48 h of treatment compared to 24 h, see Table 1.

NF-κB protein showed a very intense immunostaining in the nuclear compartment in untreated samples, while the cytoplasm was less stained, see Figure 6G–I. In treated cells, the presence of this protein was appreciable only in the cytoplasmic compartment and the staining was evenly distributed throughout the cell Figure 6J–L. The semi-quantitative evaluation of immunostaining intensity (IFIS) in Table 1 showed that the level of NRF-2 and NF-κB proteins had a decreasing trend in a dose- and time-dependent manner, with statistical significance of the different expressions among the groups of treated cells with respect to the untreated ones.

**Figure 7:**
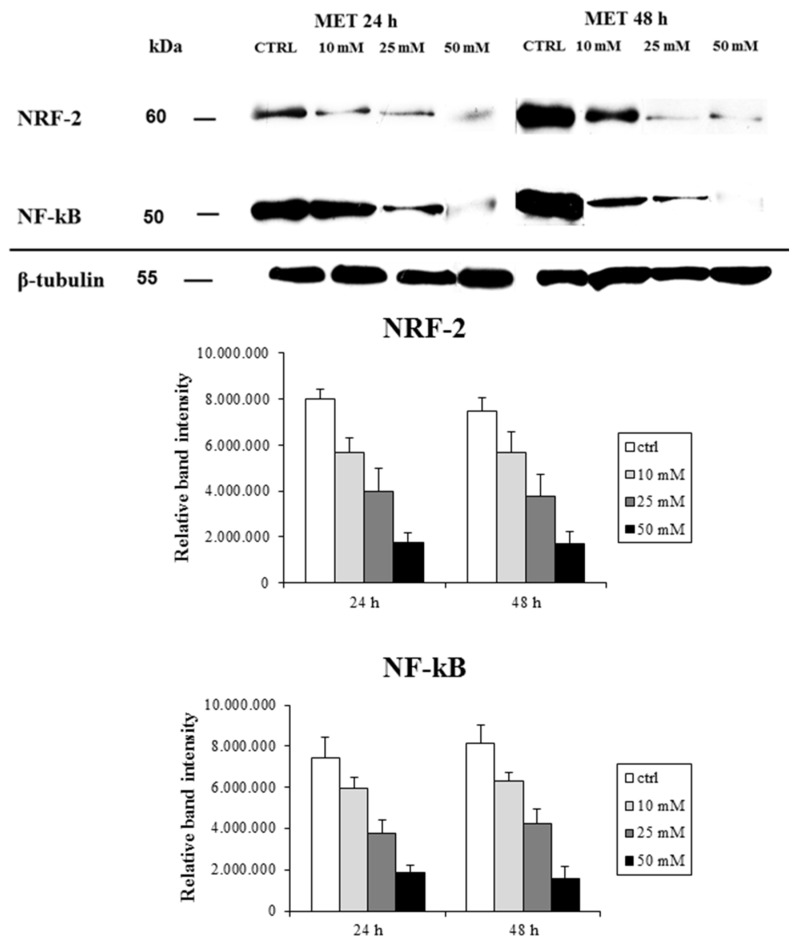
Western blot analysis of HT29 cells treated with graded concentrations of metformin, 10 mM/25 mM/50 mM for 24 h and 48 h, and untreated cells (CTRL), using anti-NRF-2 and anti- NF-κB antibodies. Mean densitometric data of NRF-2 and NF-κB expressions were analyzed using NIH ImageJ software. *p* < 0.05 between all group pairs.

Western blot analysis showed that NRF-2 expression decreased after treatment with increasing concentrations of metformin. This observation was more evident after 48 h of treatment. The expression pattern of NF-κB was slightly different, and the protein amount decreased with higher doses of metformin with respect to NRF-2, Figure 7.

Densitometric analysis and normalization (with equal amounts of protein loading) of the immunoreactivity signals from protein extracts of treated and untreated cells showed that the expression pattern had a decreasing trend related to the dose and time of treatment. Western blotting and densitometric analysis were performed in triplicate; the quantification of protein expressions between different groups of treatment and the untreated group achieved statistical significance.

### 2.3. Flow Cytometry Analysis Demonstrates That Metformin Exerts an Inhibitory Effect on HT29 Cells Survival

The ability of Metformin treatment to induce apoptosis in HT-29 cells was determined using an Annexin V-FITC assay to establish the relationship between antiproliferation and apoptosis.

**Figure 8:**
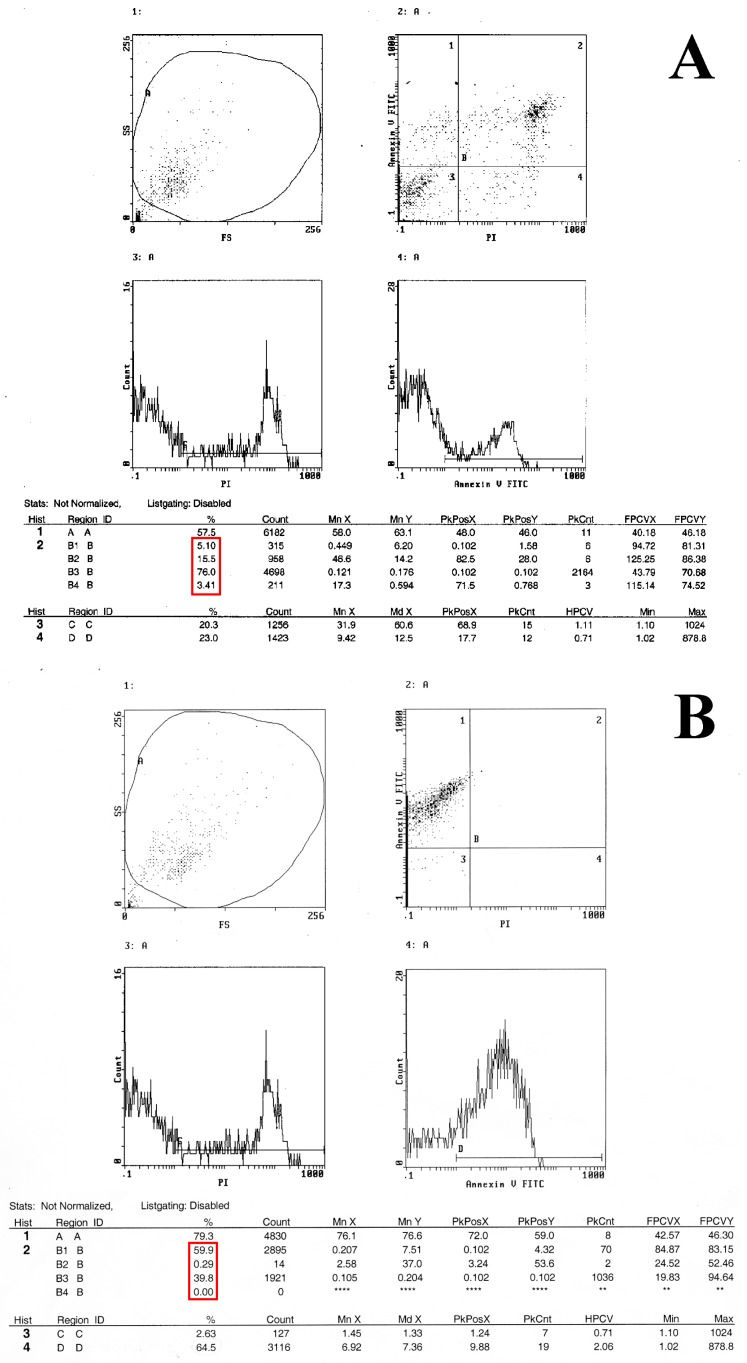
Flow cytometry analysis of untreated (**A**) and treated (**B**) cells with graded concentrations of metformin, using an Annexin V-FITC assay. (**A**) The early apoptotic death rate of untreated HT-29 cells was 5.1% (line 2, third column of the Table below the Figure). (**B**) The early apoptotic death rate of the HT-29 cells treated with MET was remarkably higher than the control. The highest rate of apoptotic cells was achieved with the 50 mM treatment for 48 h, i.e., 59.9% (line 2, third column of the Table below the Figure). In the red box are evident the apoptotic rates for untreated (panel A), and metformin treated (panel B) cells

Staining with FITC Annexin V clearly showed that the percentage of apoptotic HT29 cells treated with metformin increased in a time- and dose-dependent manner, when comparing treatments to each other. Similarly, the early apoptotic death rate of the HT-29 cells treated with MET was remarkably higher than the controls Figure 8A,B. The highest rate of apoptotic cells (i.e., 60%) was achieved with the 50 mM treatment for 48 h, Figure 8B. Flow cytometry analyses were performed in triplicate and the results, which reached statistical significance, agreed with those obtained by the confocal microscopy analysis and western blot experiments.

## 3. Discussion

In this study, HT29 cells were treated with different concentrations of metformin, 10 mM/25 mM/50 mM for 24 and 48 h. We performed immunofluorescence experiments by means of confocal microscopy and western blot analysis to evaluate a panel of factors involved in apoptotic/autophagic processes and oxidative stress response. Moreover, HT29 cells treated with metformin were analyzed by flow cytometry assay to detect the apoptotic rate of the cells. The first observations concerning the phenotype of HT29 cells treated with metformin showed modifications of cell morphology, probably due to the partial loss of plasma membrane attachment; these features are characteristic of the earliest phases of the apoptotic process. The immunofluorescence analysis of Ki-67 expression pattern corroborates this finding; in fact, there was a strong decrement of Ki-67 protein in treated cells with respect to the untreated ones. All these results were related to dose and time of treatment. Moreover, the apoptotic-inducing activities of metformin causes an increase in the early apoptotic death rate that results in poor survival of cells after treatment, as clearly demonstrated by flow cytometry experiments. Overall, these results show that metformin may not only inhibit the growth of HT29 cells, but also induces cell apoptosis. These considerations were supported by the analysis of key factors involved in the apoptotic process as APAF-1, Cleaved PARP-1, and caspase-3 active. Cell apoptosis is activated in response to both intrinsic and extrinsic pathways. Drug therapies that induce apoptosis cause DNA damage-induced and p53-regulated release of cytochrome-c from mitochondria. In turn, cytochrome-c binds to apoptosis-activating factor-1 (APAF-1), resulting in the activation of caspase 9, which is followed by the activation of effector caspases including caspase 3. Following ligation, death receptors signal cell death by inducing a death-inducing signaling complex composed of the cytoplasmic adapter protein FADD (Fas-associated death domain) and caspase 8. Activated caspase 8 can activate caspase 3 both directly and indirectly by truncation of Bid [21]. Metformin has been shown to induce cancer cell apoptosis through both the intrinsic and extrinsic pathways [22]. In this study, we observed that caspase 3 active, Cleaved PARP-1, and APAF-1 expressions were higher in treated cells with respect to untreated cells in a statistically significant manner, already in the lowest metformin concentration used (10 mmol/L). Previously, metformin at 20 mmol/L has been shown effective against breast cancer [23], melanoma [24], and gastric cancer [25]. The concentration of metformin administered to type 2 diabetic patients is approximately 30–60 μmol/L [26]. Thus, the doses of metformin that were shown effective against cancer cells are approximately 300–600 fold (around 20 mmol/L) greater than the dose routinely administered for diabetic disorders, and about 1000 times higher than what is used in common practice [27]. In this study, we used also a low dose of metformin (10 mmol/L) in order to establish whether the drug induces apoptosis at doses that avoid toxic side effects to cells. The results confirmed that metformin was effective even at low doses, whereas higher metformin concentrations, being toxic, will lead to apoptosis. Furthermore, for anticancer metformin translational research the metformin concentration obviously needs to change. Elevated levels of autophagic marker expression were observed in treated cells. This result may have controversial interpretations, in fact recent studies suggest that autophagy plays a dual role in determining cell fate, which means that it could be a survival mechanism or induce programmed cell death, depending on different cellular stresses. In some conditions, the activation of autophagy provides cell protection presumably by eliminating dysfunctional organelles and proteins and maintaining the energy balance. To a large extent, the cytoprotective function of autophagy appears to be a consequence of the fact that autophagy induction inhibits apoptosis through a cross-talk between autophagy and apoptosis regulatory pathways. On the other hand, autophagy is also considered a type of cell death program [28]. It is well known that there are 3 types of cell death. Necrosis, a type of unprogrammed cell death, evokes cellular inflammation reaction by immunological activation and subsequently leads to cell death in neighbor cells. Apoptosis, which is programmed cell death type I, is characterized by the formation of chromatin condensation, DNA fragmentation, and apoptotic bodies. Cell death by overactivated autophagy has been referred to as programmed cell death type II [29]. Upregulation of autophagy has been shown to lead to cell death in order to eliminate damaged or abnormal cells. It would seem reasonable to expect that cytotoxic autophagy should be a consequence of higher levels or a more prolonged process of cellular self-digestion rather than of cytoprotective autophagy. However, there is no explicit experimental evidence suggesting that cytotoxic autophagy is uniquely different from cytoprotective autophagy. In general, depending on the type of the tumor and stimuli, as well as the extent of DNA damage, autophagy can have both cytoprotective and cytotoxic functions [30].

Another point to be discussed is the response of NRF-2 protein to metformin treatment; the NRF-2 expression profile is negatively regulated by the drug in a dose- and time-dependent manner. This finding is very intriguing as there are researches geared towards defining the boundaries between NRF-2 positive and negative effects in cancer, which aim to establish a precise rationale for undertaking NRF-2 therapeutic targeting. The main function of the transcription factor NRF-2 is to activate the cellular antioxidant response by inducing the transcription of several genes to protect cells from the effects of exogenous and endogenous insults such as xenobiotics and oxidative stress [31]. As a result, NRF-2 has been typically regarded as a cytoprotective transcription factor and is considered as the main defense mechanism of the cell and a major regulator of cell survival. Thus, NRF-2 has been traditionally deemed to be a tumor suppressor. Indeed, NRF-2-deficient mice are more sensitive to carcinogenesis [32,33] and NRF-2 loss has been linked to enhanced metastatic potential [34,35]. However, in the past few years, increasing evidence suggest that NRF-2 activation might not be beneficial in all cancer types and stages. In fact, NRF-2 promotes survival not only of normal cells but also of cancer cells, supporting the hypothesis that NRF-2 activation in malignant cells might sustain the evolution of the disease. The identification of a ‘dark side’ of NRF-2 [36,37] has generated controversy because it is still unclear whether NRF-2 acts as a tumor suppressor or as an oncogene [36,38]. Indeed, since oxidative stress is involved in the initiation of cancer, the anti-oxidative function of NRF-2 may play an anticancer role and may be useful in cancer chemoprevention. NRF-2 hyperactivation in tumors creates an environment that may favor the survival of cancer cells by protecting them from excessive oxidative stress, chemotherapeutic agents, or radiotherapy [31,39,40]. Indeed, in human tumor samples and cell lines, constitutive NRF-2 activation increases the expression of some genes involved in drug metabolism, thereby sustaining resistance to chemotherapeutic drugs and radiotherapy [41]. NRF-2 can play an important role in chemoresistance, preventing the intracellular accumulation of drugs in cancer cells and subsequently protecting cells from apoptosis [42]. These literature data are in line with our observation that metformin induces an increase of apoptotic key-factors and reduces the nuclear translocation of NRF-2 protein. To our knowledge, this is the first study in which the effect of metformin on NRF-2 protein is investigated, and it may provide an opportunity to study new molecules capable of interfering with the binding and activation of the NRF-2 pathway. Moreover, NF-κB expression pattern is drastically affected by metformin treatment, in fact, treated cells showed a significant decrease in NF-κB protein levels. In addition, the protein was present only in the cytoplasmic compartment, whereas, in untreated cells NFκB was expressed at the nuclear level. Several lines of evidence have shown that the transcription factor, nuclear factor-kappa B (NF-κB), is a critical determinant, as it regulates a variety of pathophysiological processes, such as inflammation, cell survival, proliferation, invasion, apoptosis, differentiation, and chemo-resistance in different tumor cells, including colorectal [43,44]. Therefore, pharmacologically-safe antitumor agents with potential to influence the tumor microenvironment and inhibit NF-κB signaling activation may enhance chemo-sensitivity and reduce metastasis of tumor cells, thus providing a promising approach for the prevention or treatment of tumors. Moreover, several studies are posing NF-κB as a key regulator in the cross-talk among the pathways leading to type 2 diabetes mellitus, inflammatory bowel diseases, and colorectal cancer [20]. Actually, various carcinogens, growth factors, inflammatory stimuli including microbiota and pro-oxidants activate the transcription factor NF-κB which plays a central role in inflammation and is mostly expressed in cancers [45]. NF-κB plays a pivotal role in linking chronic inflammation to cancer development through its ability to upregulate several inflammatory and tumor promoting cytokines such as IL-6, IL-1α, and TNF-α, as well as survival genes such as *BCL2* and *BCLXL* [46]. In addition, NF-κB promotes the epithelial–mesenchymal transition (EMT), through activation of snail and twist [47] in the microenvironment, and the expression of inflammatory cytokines. Moreover, NF-κB seems to be involved in tumor-associated macrophage (TAM) recruitment and acts in cancer-associated fibroblasts (CAF), by promoting the expression of a proinflammatory gene signature, which is important for macrophage recruitment, neovascularization, and tumor growth, which are abolished when NF-κB is inhibited [48] This study shows that metformin has an antiproliferative effect related to changes in the expression of NRF-2/NF-κB pathways, as well as an apoptotic effect on human colon cancer cells. Although further studies are needed to elucidate the detailed correlation between these processes, it can be hypothesized that metformin, which is already widely used in humans as antidiabetic drug, might be a promising candidate for the prevention or treatment of colorectal cancer. On the other hand, type 2 diabetes mellitus, colorectal cancer, and inflammatory bowel diseases are commonly occurring interrelated clinical problems. They share a common basis influenced by disease-related inflammation, a process characterized by upregulation of expression of common inflammatory cytokines along with TGFβ, TNFα, NF-κB, ROS and other signaling molecules, leading to an imbalance in the intestinal microbiota. The intersection and the converging of all these molecular pathways constitute a cross-talk, which affects the pathogenesis of the above-mentioned chronic diseases. A treatment that can interfere with this cross-talk could be a novel therapeutic target of interest in managing the three diseases. The authors are aware that some aspects of the present work are still critical; these preliminary data will be refined in more sophisticated, preplanned experiments.

## 4. Materials and Methods

### 4.1. Cell Culture

The human colon carcinoma cell line HT29 was obtained from the American Type Culture Collection and was grown in Dulbecco’s modified Eagle’s medium supplemented with 10% fetal bovine serum, glutamine (4 mM), and penicillin (100 U/mL)/streptomycin (100 g/mL). Cell cultures were maintained at 37 °C in 5% CO_2_ and 95% air. HT29 cells were plated at the approximate density of 1 × 10^6^ cells/dish in 10 cm^2^ culture dishes, 4 × 10^4^ cells/well in two-well chamber glass slides, and 1 × 10^4^ cell/well in 96-well tissue culture plates. When cells reached approximately 60% confluence, the medium was replaced with serum-free cell medium containing different concentrations of metformin (0, 10, 25, and 50 mmol/L) and the cells were cultured for 24 and 48 h at 37 °C in 5% CO_2_ and 95% air. The cells were trypsinized with 0.25% trypsin and 0.2 g/L EDTA, and harvested for further studies. Metformin was purchased from Sigma Aldrich (Saint Louis, MO, USA; PHR 1084) and was dissolved in sterile water at a stock dose of 1 M for further application.

### 4.2. Cell Counting

Cells were seeded at a density of 20,000/well and were counted at day one, two, and three after plating. For counting, cells were detached from the wells by washing in a 0.25% trypsin and 0.2 g/L EDTA solution. Thereafter, 1 mL normal culture medium was added to stop the trypsinization; a 500 µL cell suspension, to which 70 µL trypan blue was added, then, was transferred into a Burker chamber for cell counting. The number of cells, expressed as number/mL, was determined by two blinded observers.

### 4.3. Western Blot Analysis

Whole cell lysates were obtained from cultured cells, as described above, and extracted with hypotonic buffer (50 mM Tris-Cl, pH 7.8, containing 1% Nonidet P40, 140 mM NaCl, 0.1% SDS, 0.1% Na deoxycholate, 1 mM Na_3_VO_4_, and freshly added protease inhibitor cocktail). Lysates were then cleared by centrifugation for 15 min in a refrigerated centrifuge at maximum speed, and then were immediately boiled in SDS sample buffer. Forty milligrams of protein extract from each treatment condition was electrophoresed on SDS-PAGE and transferred to nitrocellulose membranes. The membranes were blocked with 3% dry milk and 2% BSA in PBS-T, and incubated with the following antibodies (diluted 1:1000 overnight at 4 °C under agitation), mouse anti-human APAF-1, rabbit anti-human MAPLC3, mouse anti-human NF-κB (Santa Cruz), mouse anti-human PARP-1 cleaved (Cell Signaling, Beverly, MA, USA), mouse anti-human NRF-2 (Abcam, Cambridge, UK), and mouse anti-human Ki-67 (DAKO, Glostrup, Denmark). After washing, the membranes were incubated with secondary HPR-conjugated goat anti-mouse IgG antibody (1:5000) or HPR-conjugated goat anti-rabbit IgG antibody (1:5000) (Thermo scientific, Waltham, MA, USA) for 30 min at room temperature. Immunoreactive proteins were detected with ECL (Amersham, Little Chalfont, UK). Anti-mouse-β tubulin (Sigma Aldrich) was used as loading control. Densitometry analysis was performed using a KODAK (Rochester, NY, USA) Image Station 440 cf system), and semi-quantitative analysis was performed with NIH Image J software. For each sample and marker, the band intensities were normalized to β-tubulin, and results were expressed as the normalized treatment to control ratio.

### 4.4. Evaluation of Immunofluorescence by Confocal Microscopy

Cells were cultured in two-well chamber glass slides which were used for immunofluorescence analysis to evaluate the expression of MAPLC3, APAF-1, PARP-1 cleaved, NRF-2, NF-κB, and Ki-67 proteins. Monolayered cells were fixed in 4% paraformaldehyde in PBS for 10 min. After a treatment with 3% BSA in PBS for 30 min at room temperature, they were incubated with the primary antibodies (mouse anti-human APAF-1, rabbit anti-human MAPLC3, mouse anti-human caspase-3 active, mouse anti- human NF-κB, Santa Cruz mouse anti-human PARP-1 cleaved, mouse anti-human NRF-2, Abcam mouse anti-human Ki-67, and DAKO), and diluted 1:25 in PBS containing 3% BSA for 1 h at room temperature. After washing in PBS, the samples were incubated for 1 h at room temperature with the secondary antibodies diluted by 1:20 in PBS containing 3% BSA (sheep anti-mouse FITC conjugated, goat anti-rabbit TRITC conjugated, Sigma Aldrich). After washing in PBS and in H_2_O, the samples were counterstained with 1 mg/mL DAPI in H_2_O and then mounted with antifading medium (0.21 M DABCO and 90% glycerol in 0.02 M Tris, pH 8.0). Negative control samples were not incubated with the primary antibody. The confocal imaging was performed under a Leica TCS SP2 AOBS confocal laser scanning microscope. Excitation and detection of the samples were carried out in sequential mode to avoid overlapping of signals. Sections were scanned with laser intensity, confocal aperture, gain, and black level settings kept constant for all samples. Optical sections were obtained at increments of 0.3 mm in the *z*-axis and were digitized with a scanning mode format of 512 × 512 or 1024 × 1024 pixels and 256 grey levels. The confocal serial sections were processed with the Leica LCS software to obtain three-dimensional projections. Image rendering was performed by Adobe Photoshop software. The original green fluorescent confocal images were converted to greyscale and median filtering was performed. An intensity value between 0 (black) and 255 (white) was assigned to each pixel. Background fluorescence was subtracted and immunofluorescence intensity (IF) was calculated as the average for each selected area. The fluorescence intensity at the selected areas, linearly correlated with the number of pixels, and was quantitatively analyzed using the standard imaging analysis software of an NIS-Elements System. Each sample was assigned a code number and score, referred to as the Immunofluorescence Intensity Score (IFIS), which was determined by an observer who was blind to tissue groups during the analysis [49].

### 4.5. Flow Cytometry Analysis

The apoptotic status of HT-29 cells was evaluated by measuring the exposure of phosphatidylserine on the cell membranes using Annexin V-fluorescein isothiocyanate (Annexin V-FITC) and propidium iodide (PI) staining [50]. The BD Pharmingen Annexin V-FITC Apoptosis Detection Kit I (BD Biosciences, Frankrin Lakes, NJ, USA) was used for the apoptosis assay. HT-29 cells were plated in a 24-well plate (1 × 10^6^ cells mL^−1^), and after a 24 h incubation, the cells were treated with graded concentrations of metformin (0, 10, 25, and 50 mmol/L) for 24 and 48 h, and harvested. After centrifugation, the cell pellets were washed twice with cold phosphate-buffered saline (PBS: 137 mM NaCl, 2.7 mM KCl, 10 mM Na_2_HPO_4_, pH 7.4) and suspended in 100 µL of 1× binding buffer (10 mM Hepes/NaOH, 140 mM NaCl, 2.5 mM CaC_l2_, pH 7.4). The cells were incubated with 5 µL Annexin V-FITC and 10 µL PI at room temperature for 15 min in the dark. After the incubation, 400 µL of 1× binding buffer was added to each tube. The cells were analyzed immediately by Epics XL-MCL Flow Cytometry (Beckman Coulter, Cassina de Pecchi, Italy).

### 4.6. Statistical Analysis

All experiments were performed in triplicate. Data were expressed as means ± standard deviation. Statistical comparisons of results were made using analysis of variance (ANOVA). Significant differences (*p* < 0.05) between the means of control and metformin treated cells were analyzed by a Student’s *t*-test.

## Figures and Tables

**Table 1:** Evaluation of immunostaining intensity (Immunofluorescence Intensity Score: IFIS).

Proteins	IFIS (Mean ± SD)-24 h				IFIS (mean ± SD)-48 h			
	CTRL	10 mM	25 mM	50 mM	CTRL	10 mM	25 mM	50 mM
**PARP-1**	20.0 ± 4.3	47.0 ± 11.0	63.0 ± 9.8	76.0 ± 15.0	23.0 ± 12.0	55.0 ± 6.7	74.0 ± 13.0	89.0 ± 22.0
**Caspase-3**	36.0 ± 9.5	49.0 ± 23.0	69.0 ± 15.0	91.0 ± 7.4	32.0 ± 5.7	64.0 ± 17.0	97.0 ± 20.0	101.0 ± 29.0
**APAF-1**	31.0 ± 7.5	54.0 ± 3.9	66.0 ± 19.0	85.0 ± 9.5	27.0 ± 7.0	72.0 ± 17.0	72.0 ± 17.0	93.0 ± 12.0
**MAP-LC3**	26.0 ±4.7	45.0 ± 7.2	71.0 ± 7.6	93.0 ± 15.0	28.0 ± 8.3	57.0 ± 6.3	84.0 ± 5.4	103.0 ± 32.0
**NRF-2**	55.0 ± 7.2	43.0 ± 6.3	31.0 ± 4.8	19.0 ± 3.7	63.0 ± 12.0	47.0 ± 5.4	30.0 ± 7.5	23.0 ± 3.0
**NF-κB**	60.0 ± 9.5	54.0 ± 8.3	47.0 ± 15.0	32.0 ± 7.4	67.0 ± 5.7	50.0 ± 17.0	33.0 ± 2.0	25.0 ± 2.9

*p* < 0.05 between all group pairs.
